# Genic insights from integrated human proteomics in GeneCards

**DOI:** 10.1093/database/baw030

**Published:** 2016-03-28

**Authors:** Simon Fishilevich, Shahar Zimmerman, Asher Kohn, Tsippi Iny Stein, Tsviya Olender, Eugene Kolker, Marilyn Safran, Doron Lancet

**Affiliations:** ^1^Department of Molecular Genetics, Weizmann Institute of Science, Rehovot, 7610001, Israel; ^2^LifeMap Sciences Ltd., Tel Aviv 69710, Israel; ^3^CDO Analytics, Seattle Children’s Hospital, Seattle, WA 98101 USA; ^4^Bioinformatics and High-Throughput Analysis Laboratory, Seattle Children’s Research Institute, Seattle, WA 98101 USA; ^5^Data-Enabled Life Sciences Alliance (DELSA), Seattle, Washington, 98101, USA; ^6^Departments of Biomedical Informatics and Medical Education and Pediatrics, University of Washington School of Medicine, Seattle, WA 98109, USA; ^7^Department of Chemistry and Chemical Biology, Northeastern University College of Science, Boston, MA 02115 USA

## Abstract

GeneCards is a one-stop shop for searchable human gene annotations (http://www.genecards.org/). Data are automatically mined from ∼120 sources and presented in an integrated web card for every human gene. We report the application of recent advances in proteomics to enhance gene annotation and classification in GeneCards. First, we constructed the Human Integrated Protein Expression Database (HIPED), a unified database of protein abundance in human tissues, based on the publically available mass spectrometry (MS)-based proteomics sources ProteomicsDB, Multi-Omics Profiling Expression Database, Protein Abundance Across Organisms and The MaxQuant DataBase. The integrated database, residing within GeneCards, compares favourably with its individual sources, covering nearly 90% of human protein-coding genes. For gene annotation and comparisons, we first defined a protein expression vector for each gene, based on normalized abundances in 69 normal human tissues. This vector is portrayed in the GeneCards expression section as a bar graph, allowing visual inspection and comparison. These data are juxtaposed with transcriptome bar graphs. Using the protein expression vectors, we further defined a pairwise metric that helps assess expression-based pairwise proximity. This new metric for finding functional partners complements eight others, including sharing of pathways, gene ontology (GO) terms and domains, implemented in the GeneCards Suite. In parallel, we calculated proteome-based differential expression, highlighting a subset of tissues that overexpress a gene and subserving gene classification. This textual annotation allows users of VarElect, the suite’s next-generation phenotyper, to more effectively discover causative disease variants. Finally, we define the protein–RNA expression ratio and correlation as yet another attribute of every gene in each tissue, adding further annotative information. The results constitute a significant enhancement of several GeneCards sections and help promote and organize the genome-wide structural and functional knowledge of the human proteome.

**Database URL**: http://www.genecards.org/

## Introduction

Achieving deep quantitative coverage of proteomes is an essential milestone in the characterization of the human organism, its tissues and its genes. Technological advancements in the past decade have promoted high-throughput shotgun mass spectrometry (MS) proteomics towards the ability to quantify the whole proteome of a given sample (1). The Human Proteome Project (HPP) is a world-wide collaborative program aimed at disseminating proteomic technologies and better integrating them with other high-throughput approaches (2). The chromosome-based HPP specifically focuses on expanding our understanding of chromosomes and protein-coding genes (3). In parallel with this work, two comprehensive human proteome studies provided significant genome-wide coverage for a large number of tissues (4, 5) (see Discussion).

A variety of online resources have been built to store, process and integrate proteomics data. Some sources store raw data files, which are not readily available for use without in-house processing (6, 7). A few online sources perform integration and standardization of data, making the processed results available to the community [ProteomicsDB (5), Multi-Omics Profiling Expression Database (MOPED) (8), Protein Abundance Across Organisms (PaxDb) (9), The MaxQuant DataBase (MaxQB) (10) and Human Proteome Map (4)]. Each data source reflects a partial picture, adopting unique methodology and parsing different content, which makes integration a necessity.

GeneCards, the human gene compendium, is a gene-centric database integrating data for 152 704 human genes from 125 sources (11). Some data in GeneCards, such as gene aliases and summaries, are straightforward to interpret. Other data are analyzed after the mining steps, in order to improve its presentation and create new scientific insights. For example, unification of 3215 pathways from 12 sources into 1073 SuperPaths provides a comprehensive view of the pathways realm (12). This was done not only by collecting data mined from multiple sources but also via judicious integration, reducing redundancy and optimizing the level of pathway-related informativeness for individual genes.

Taking advantage of the great advances in quantitative proteomics, and using GeneCards’ integration philosophy, we aimed to unify, analyze and leverage the main sources of human proteomics data, making them readily available to users via GeneCards gene annotation. The crux of this is the construction of HIPED (the Human Integrated Protein Expression Database), a unified one-stop shop, gene-centric view of the protein expression realm. HIPED data supplies novel gene annotations. Its expression proteomics fingerprint enables the creation of a metric for genes comparisons. Gene pairs with highest similarity are annotated as expression partners, creating a novel gene network. Additionally, in order to strengthen the tissue annotation of genes in GeneCards, we calculated the set of tissues for each gene in which it is differentially expressed.

Gene expression studies were previously used as the basis for a variety of gene annotations and functional insights. This includes determination of gene–gene expression-wise relations (13, 14), and definition of gene sets with specific expression profiles such as tissue specificity and housekeeping properties (15 –17). One of the most popular databases of protein–protein interactions (PPIs), STRING (18), includes microarray coexpression scores as a part of the PPI pipeline. As most previous studies were based on RNA-based datasets, the data in HIPED allow a proteomics-based look, in search for novel gene annotations and classifications.

HIPED enables the characterization of protein and RNA relations for human genes. The relationships between RNA and protein levels are generally far from being straightforward (19, 20). The ability to predict protein abundance from RNA levels is made complex because of the contribution of diverse regulatory processes controlling the transcription, processing and degradation of mRNAs and the translation, localization, modification and destruction of proteins (20, 21). Most studies focus on comparisons of protein to RNA content of a given cell/tissue (22), only few concentrate on the genes across-tissue protein–RNA expression vectors (5, 23). HIPED enables comprehensive comparisons, which can assist in gene characterizations, further facilitating the discovery of specific gene properties and mechanisms affecting protein–RNA relationships.

## Methods

### Mining and integrating human proteomes

Protein expression sources provided data either via txt files (PaxDb version 3.0 integrated datasets, MOPED version 2.5, MaxQB 09/2013) or via API methods (ProteomicsDB 02/2015). A standard symbolization algorithm was developed to map all identifiers used [sources gene identifiers, Ensembl (24) and UniProt (25) protein identifiers] to gene symbols, using GeneCards aliases, identifiers and protein annotations. For genes having multiple proteins, expression data were summed to receive a gene centric aggregated value. For proteins belonging to multiple genes, protein intensity values were equally divided among such genes.

For integration, we converted all abundance data to mole-based parts per million (PPM), whereby for MaxQB and ProteomicsDB, iBAQ (intensity based absolute quantification) was converted to PPM by

$${PPM}_{i}=\frac{{iBAQ}_{i}}{\sum_{j}{iBAQ}_{j}}\cdot 10^{6}$$

Duplicate samples and samples having <10 mapped genes were excluded from HIPED. This resulted in the definition of 771 proteomic samples (Supplementary Table S1). Of these, 555 samples represented cell lines and disease-related datasets, and 216 samples were normal human anatomical entities (tissues, *in vivo* cells and body fluids). Experimental data of individual samples independently curated by different sources were pre-averaged (Supplementary Table S4). Further averaging across data sources (see Supplementary Table S2 and legend), we ended up with 69 datasets for normal anatomical entities (Supplementary Table S3) and 125 for the cell lines (Supplementary Table S1). Averages were calculated as geometric mean of non-zero PPM values. Three datasets with extremely high gene counts (>12 000; Supplementary Figure S5 outliers, proteomes #15, 17, 18 in Supplementary Table S2) were excluded from averaging.

### Mining and integrating human transcriptome

GTEx (26) RPKM (reads per kilobase of transcript per million mapped reads) values for similar anatomical entities were averaged across all individuals, resulting in 51 averaged samples (Supplementary Table S13). Further, we additionally averaged sample types of very close anatomical sources (e.g. coronary artery and aorta) to produce another seven averaged samples, to a total of 58 averaged RNA-seq datasets. This data were incorporated in GeneCards RNA expression histogram images and used in the protein versus RNA comparative analyses.

Differential expression was computed using the DESeq2 package (27), where each tissue was tested against all tissues. Genes with maximal cross-tissue average read count <5 were not used. As previously reported (28), the DESeq package was not readily scalable for such a large dataset (total of 2712 samples). Therefore, the GTEx read counts for similar anatomical entities were pre-averaged across all individuals for differential expression calculations. We compared this alternative approach with using the DESeq built-in replicate handling. Three sample analyses were performed on one tissue versus seven controls (Supplementary Figure S19). Analyzing the fold change values for all 35 514 genes, the average of Pearson correlation value was 0.82, suggesting good agreement between the methods. Further, comparing differential expression annotation with identical cutoff of X4, the average false-positive rate was 1.0%, whereas the false-negative rate was 2.3%, suggesting that the alternative method is somewhat more conservative in assigning differential expression to genes.

### Analysis of protein expression

Unless otherwise stated, the protein abundance PPM values were log_10_ transformed and right-shifted into positive numerical space. The shift value was determined as log_10_ of dataset-wide minima divided by 2. Proteomics-based fold change values were calculated by dividing protein abundance of a gene in a tissue by the average protein abundance of the gene. Fold change values deriving from abundance values lower than 0.1 PPM were ignored. The expression fold change cutoff was selected on the basis of seeking an optimum between disproportionately increasing the number of overexpressed genes per tissue and excessively diminishing the number of genes remaining without tissue overexpression annotation (Supplementary Figure S15). Expression breadth was defined as number of tissues for which a gene has a non-zero abundance value.

### Gene–gene expression correlation

Pearson’s correlation coefficient was used to measure expression similarity between genes. Alternative correlation methods produced similar results to those shown in the main text using Pearson’s coefficient. Randomized tissue expression vectors were calculated by permuting the tissue expression vector of every gene. The pairwise ‘AND’ metric was calculated by counting the number of anatomical entities in which both genes have non-zero abundance values.

### Analysis of protein and RNA expression

Cellular copy numbers for both protein and RNA were computed as follows: RPKM values and PPM values were each multiplied by a factor for this unit conversion. For protein, we used a factor of 10^4^ to convert PPM to copy number, assuming 10^10^ molecules/cell (29). For RNA, we used a factor of 1, based on the assumption that one transcript copy per cell corresponds to between 0.5 and 5 FPKM (fragments per kilobase of transcript per million mapped reads) values (30) and taking an approximate geometrical mean.

For the correlation between protein and RNA expression tissue vectors, gene vectors of each tissue were log_10_ transformed, *z*-scored and right-shifted into positive numerical space, before measuring the Pearson’s correlation. In silico genes with permuted protein vectors were used as random controls. Averaged P/R ratios were calculated as the geometric mean of across-tissues P/R cell copy number ratios of a gene. P/R values having zero in either one of the parameters were ignored and genes with a 24-length zero vector were excluded.

### Bioinformatics analyses

Gene symbols, gene categories, gene-protein mappings, gene ontology (GO) terms, String PPIs (18), COMPARTMENTS cellular localization (31) and paralogs (24) were retrieved from GeneCards V4 (11); Gene–disease relationships from MalaCards V1.08 (32); Pathways gene lists from PathCards (12). Gene set enrichment analysis was performed using GeneAnalytics (33, 34).

### Tissue clustering

All computations below used the Matlab^®^ software (version R2012b; MathWorks Inc.). For hierarchical clustering, we used either 1 minus Pearson’s correlation or Jaccard coefficient as distance, and using the Ward method. K-means was performed using Pearson’s correlation as the distance metric, with 10 repeats. In clustering and principal component analysis (PCA) analyses of data from multiple sources (Supplementary Figures S4–S6), only genes reported by all sources in an analysis were used to minimize source-related bias.

### Data availability

HIPED-derived data incorporated into the GeneCards database is available upon request, via the GeneCards online ‘contact us’ form. For retrieval of raw proteomics data used to create HIPED, please contact our sources.

## Results

### Mining and integration of human proteome data

**Figure 1.**
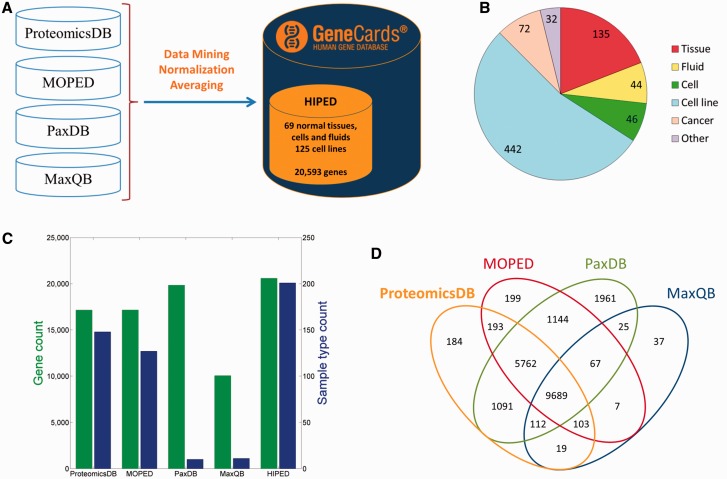
HIPED—Human Integrated Protein Expression Database. (**A**) HIPED architecture scheme. (**B**) Classification of the 771 proteomes in HIPED. (**C**) Gene and sample type counts of HIPED and its mined components. Sample types are unique normal anatomical entities or cell lines represented in each source. (**D**) Gene content overlap of HIPED mined sources.

We created HIPED, a Human Integrated Protein Expression Database (Figure 1A). HIPED encompasses 20 593 unique genes present in 771 proteome samples (Figure 1B; Supplementary Table S1). These data are mined from four proteomics sources—ProteomicsDB (5), MOPED (8), PaxDb (9) and MaxQB (10), each using different post-MS analyses on in-house and external raw MS data (Table 1). HIPED has larger gene count and sample coverage than its integrated components (Figure 1C and D), thus representing a useful unification and affording a one-stop shop for gene-centric protein expression information. HIPED data cover 19 370/21 965 (88.2%) of the genes annotated as protein coding in GeneCards (11), notably annotating also 1223 genes of other gene categories, including 458 RNA genes and 717 pseudo-genes (Supplementary Figure S1). Of the total 20 593 genes represented by HIPED proteins, 16 900 appear in normal anatomical entities (next section) and the remaining 3693 are seen only in disease tissues or in cell lines.

**Table 1.** HIPED sources.

Database	Sample count	Gene count	Database url	Reference
ProteomicsDB	577	17,153	https://www.proteomicsdb.org/	(5)
MOPED	172	17,164	http://moped.proteinspire.org	(8)
PaxDB	11	19,851	http://pax-db.org/#!home	(9)
MaxQB	11	10,059	http://maxqb.biochem.mpg.de/mxdb/	(10)

**Figure 2.**
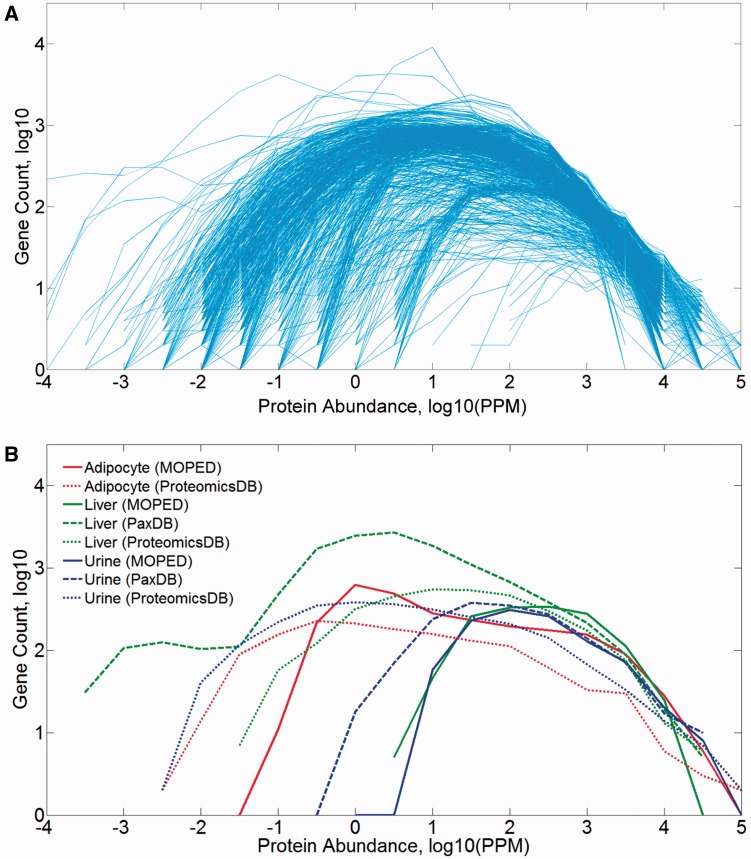
Protein abundance distribution (PPM values) of mined datasets in HIPED. (**A**) All 771 samples comprising HIPED. (**B**) Selected sample groups of similar anatomical entities.

The 771 proteome samples that include tissues and isolated cells of different types show a broad range of gene counts (83–18 756), and a diversity of log-normal abundance distributions spanning three to nine orders of magnitude (Figure 2). The distribution parameters are somewhat correlated to the number of identified proteins in the sample (Supplementary Figure S2A), and it appears that most of the observed breadth variation is related to the variance in the distribution’s minimum (Supplementary Figure S2B). A combination of experimental parameters, sample anatomical identity and pipeline processing likely contributes to such variance.

With the aim of enhancing the derivation of gene-related biological insight from proteomic data, we focused on 216 proteome samples stemming from normal human anatomical entities (tissues, *in* *vivo* cells and body fluids), to the exclusion of 555 samples which include cell lines and disease-related datasets. All further proteome analyses (except Supplementary Figure S8) were performed using the normal anatomical entities (Supplementary Tables S2 and S3). The relevant protein expression vectors were analyzed for mutual pairwise correlation, as exemplified in Supplementary Figure S3. Subsequently, hierarchical clustering was performed, which brings together tissues with similar expression vectors. In some cases, the clusters that form are related to samples that are obviously related to each other anatomically, such as different blood cells, whereas, in quite a few other cases, clusters do not obey such a rule (Supplementary Figure S4). A parallel PCA showed similar imperfect trends with respect to demarcation of broad sample types and tissue groups (Supplementary Figure S5).

**Figure 3.**
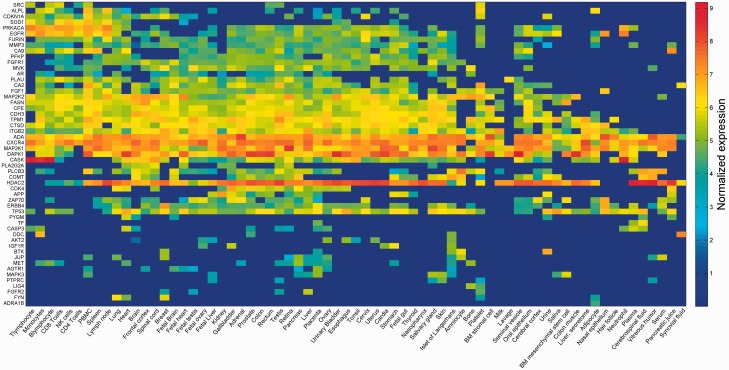
Averaged protein expression vectors. Representation of selected 53 genes averaged protein abundance vectors for the 69 anatomical entities in HIPED.

The strongest inter-vector correlation was observed for pairs of data sets arising from the exact same sample, but analyzed and curated by different pipelines (Supplementary Figures S3 and S6). This is corroborated by the high degree of overlap seen in the assignment of expressed/not expressed status for different proteins (Supplementary Table S4). Such results indicate that different modes of analysis are decidedly comparable. Lower but significant correlation values were observed for pairs of different samples from the same tissue of origin (Supplementary Figure S6). In view of such trends, and in order to characterize every anatomical entity through its pattern of protein expression, we generated a unified view by defining gene expression vectors for each of the 69 entities. These were derived from the 216 samples by averaging across different proteomic dataset representations for each of the entities (Figure 3; Supplementary Figure S7). These 69 across-genome gene vectors may be perceived as portraying a broadly disposed proteomic representation of human tissues. Interestingly, when expression vectors for tissues sampled from the group of 69 were compared with those of the respective cognate cell lines, a surprising result obtained (Supplementary Figure S8). It appears that cell lines, irrespective of their tissue of origin, tend to cluster together, often away from the cognate normal tissue (see ‘Broad tissue set’ in the Discussion).

### Proteomic tissue fingerprints for human genes

**Figure 4.**
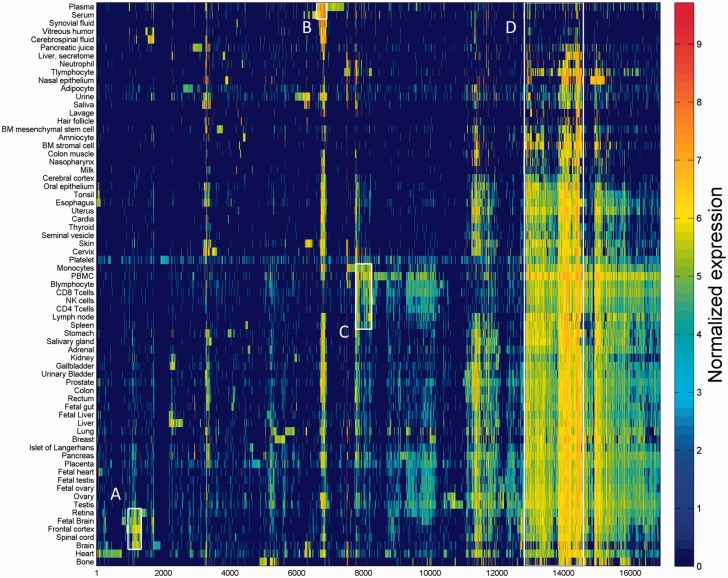
Double hierarchical clustering of the 16 900 genes in 69 normal anatomical entities. Examples of gene groups sharing functional annotations are highlighted. (A) CNS—397 genes enriched with diseases as schizophrenia, pathways as neuroscience and GO terms as transporter activity. (B) Blood—301 genes enriched with diseases such as obesity and C2 deficiency and GO terms as complement activation (C) Immune system—483 genes enriched with diseases such as rheumatoid arthritis and pathways as lymphocyte signaling. (D) Genes with housekeeping properties—1771 genes enriched with pathways and GO terms related to metabolism and gene expression. See Supplementary Tables S5–S8 for the full enrichment analysis data.

**Figure 5.**
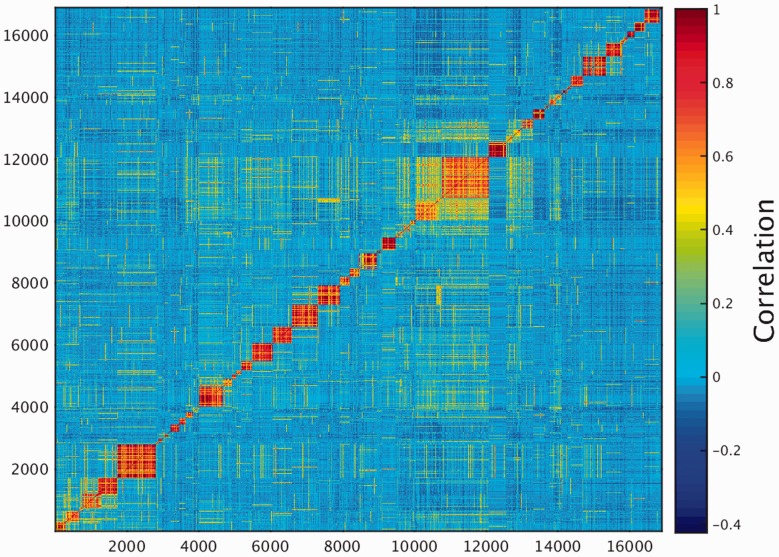
K-means analysis 53 clusters of the 16 900 genes in normal human proteomes.

One of the key outcomes of generating a large database of protein expression values across numerous genes and tissues is an emerging capacity to define an across-tissue protein abundance vector for every gene, which may be perceived as characterizing a gene’s proteomic tissue fingerprint. For this, we represented each of the 16 900 genes by a 69-dimensional vector of protein abundance across the anatomical entities (Figure 4). We then submitted the 16 900 vectors to a k-means procedure, in order to identify clusters of across-tissue expression pattern similarity. Following silhouette optimization, 53 clusters were identified (Figure 5; Supplementary Figure S9, Tables S9 and S10). Each cluster was then represented by a center-of-mass vector (Supplementary Figure S10A), which conveys tissue expression idiosyncrasies shared by genes within the cluster, often jointly including well-studied genes and genes of limited functional annotation. This procedure enables new insights on relatively uncharacterized genes (Supplementary Figure S10B).

**Figure 6.**
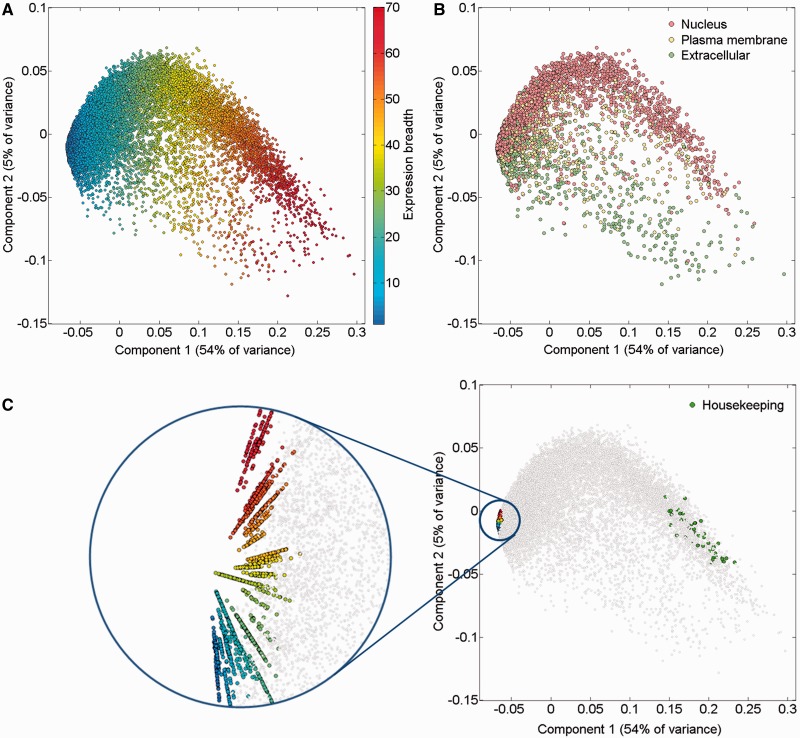
PCA of 16 900 genes comprising HIPED normal proteomes. (**A**) Gene expression breadth. Expression breadth is one of the gene expression vector signatures determining its position in the PCA space. This feature is closely related to the first component of the PCA. (**B**) Subcellular localization. Subcellular localization data from COMPARTMENTS (31) was projected on the gene expression space. Only genes having the maximal confidence score of 5 for a single subcellular compartment are shown. (**C**) Single tissue and housekeeping genes. All 2320 genes expressed in a single anatomical entity are shown, representing the tissue-specific dimensions in the expression space (left panel, different colors are used to distinguish tissues). Genes with housekeeping properties populate a specific area in the PCA space (right panel). Top 50 genes were selected with the highest pairwise similarity of across-tissue protein abundance patterns of a gene against an in silico ‘ideal’ housekeeping profile (similar expression of 10 000 PPM across all tissues and cells and 0 PPM across fluids).

**Figure 7.**
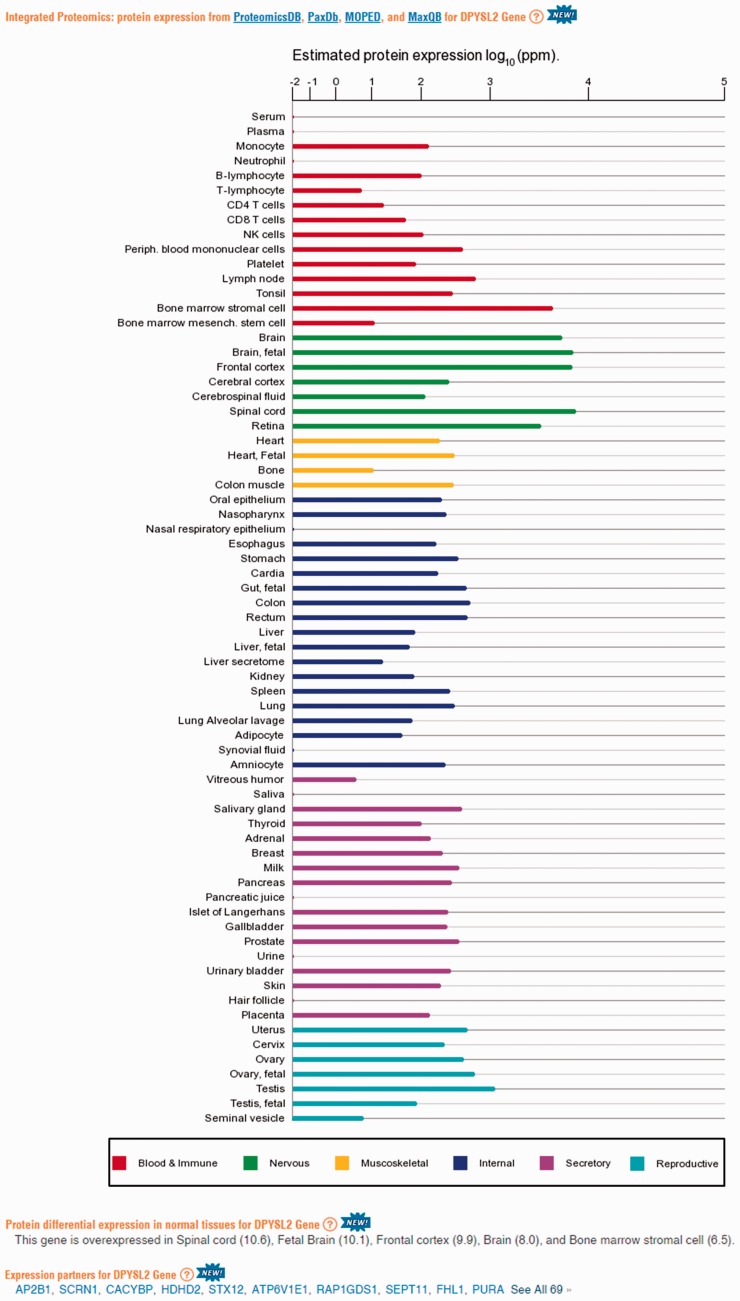
Protein abundance data in GeneCards. A screenshot of protein expression based data for the gene DPYSL2, including (i) protein expression chart; (ii) a list of tissues in which the gene is differentially expressed; (iii) a list of the gene expression partners. DPYSL2 plays a role in neuronal development and polarity, as well as in axon growth and guidance, neuronal growth cone collapse and cell migration (25). Protein expression charts are created via GeneCards automated expression charts pipeline. In order to optimize user perception of the expression, values were displayed using a special root scale (35). This scale enables viewing many orders of magnitude like on a logarithmic scale, but preserves certain characteristics of a linear scale in which the differences increase with the orders of magnitude.

An alternate way to view the protein expression space is PCA. It appears that while the first component of this PCA analysis is related to expression breadth (the number of tissues showing non-zero expression, Figure 6A; Supplementary Figure S11), the second component is related to anatomical entities identification (Figure 6C). Functional signatures, such as subcellular localization, define further characteristics of the expression space (Figure 6B). Two extremes of gene expression breadth, namely genes expressed in a single entity, and genes expressed in the majority of entities (housekeeping), populate specific areas in the expression space (Figure 6C). A visual display of protein abundance values across all 69 anatomical entities from HIPED was implemented in the expression section of GeneCards (11, 35), making the data available for scrutiny, and enabling quantitative comparisons of protein expression in every relevant gene (Figure 7). This is done in conjunction with the display of several gene expression sources, allowing facile comparison.

### A tissue expression comparison metric

**Figure 8.**
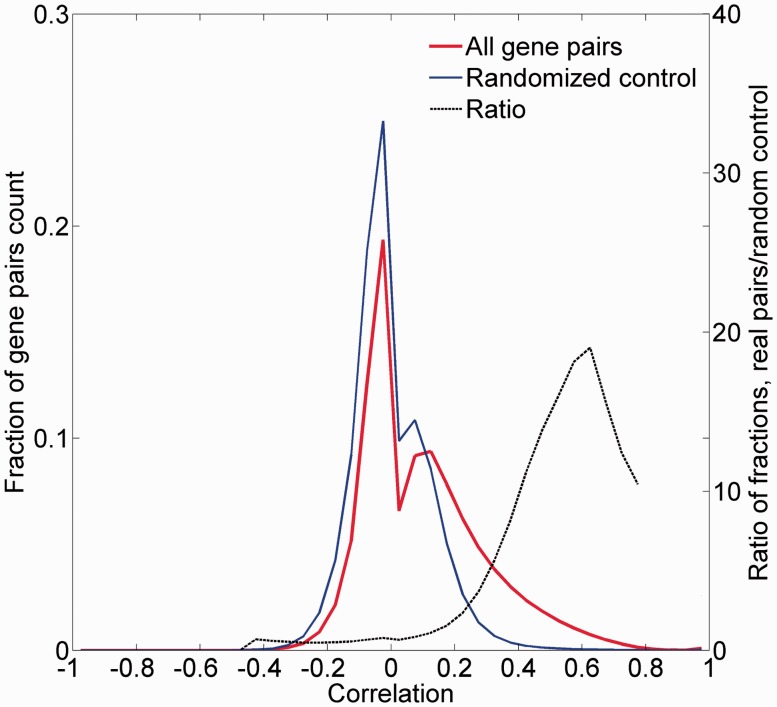
Pairwise correlation distribution. The fraction distribution of the pairwise Pearson’s correlation coefficients for the 16 900 proteome-annotated genes is plotted along random generated genes. The ratio between compared fractions distributions was plotted, disregarding bins with extremely low (<8 × 10^−5^) fraction values. Real data vectors exhibit significantly different (positively) correlation values than the random controls (Wilcoxon rank sum tailed test, *P* < 10^−5^).

We examined the legitimacy of using the correlation between tissue expression vectors as a measure of gene-to-gene similarity. For this, the distribution of all pairwise Pearson’s correlation coefficients for the 16 900 proteome-annotated genes was computed (Figure 8; Supplementary Figure S12). Control randomized vectors yielded a significantly different distribution of correlation values (*P* < 10^−^^5^). Further, there was an appreciable enrichment of test versus control counts in positive correlation values, peaking at *R* = 0.6. This suggests that the expression proximity metric we have defined can be used to assess gene-to-gene similarity.

**Figure 9.**
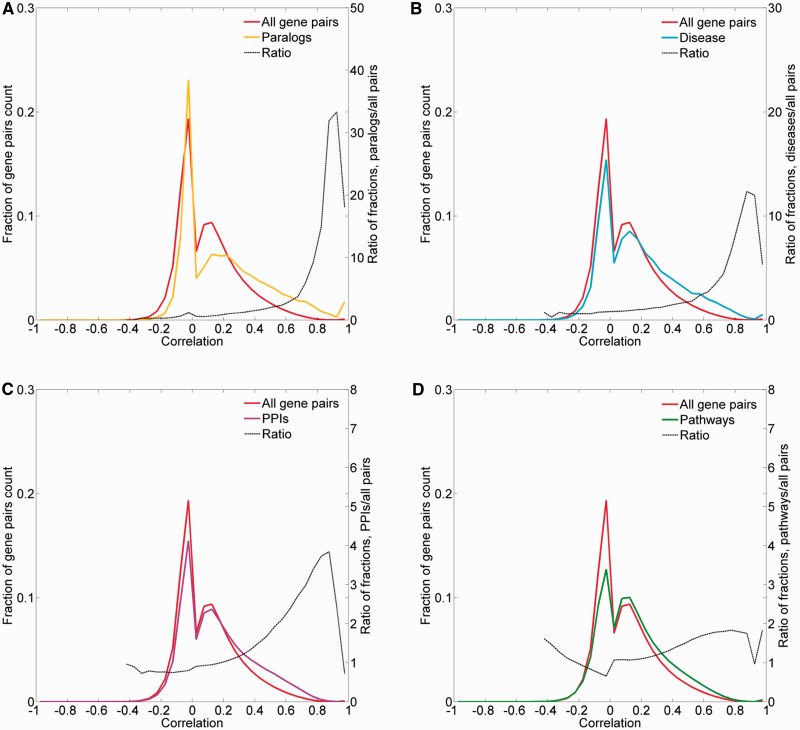
Pairwise correlation distribution. The fraction distribution of the pairwise Pearson’s correlation coefficients for the 16 900 proteome-annotated genes is plotted along gene pairs sharing functional attributes, namely: (**A**) sequence paralogs, (**B**) diseases, (**C**) PPIs and (**D**) biological pathways. The ratio between compared fractions distributions was plotted, disregarding bins with extremely low (<8 × 10^−5^) fraction values.

To obtain further evidence for the validity of the expression metric, we examined the expression correlation behavior of gene pairs sharing functional attributes: sequence paralogs, diseases, PPIs and biological pathways. In three of the cases, there was an enrichment of gene pairs with high expression similarity (*R* > 0.5) among functionally related gene pairs (Figure 9). In the case of paralogy, we further show a weak but significant correlation between protein sequence similarity and expression similarity (Pearson’s correlation coefficient = 0.39, *P* < 10^−^^3^; Supplementary Figure S13). In the case of biological pathways, the enrichment for expression-correlated gene pairs was negligible.

**Figure 10.**
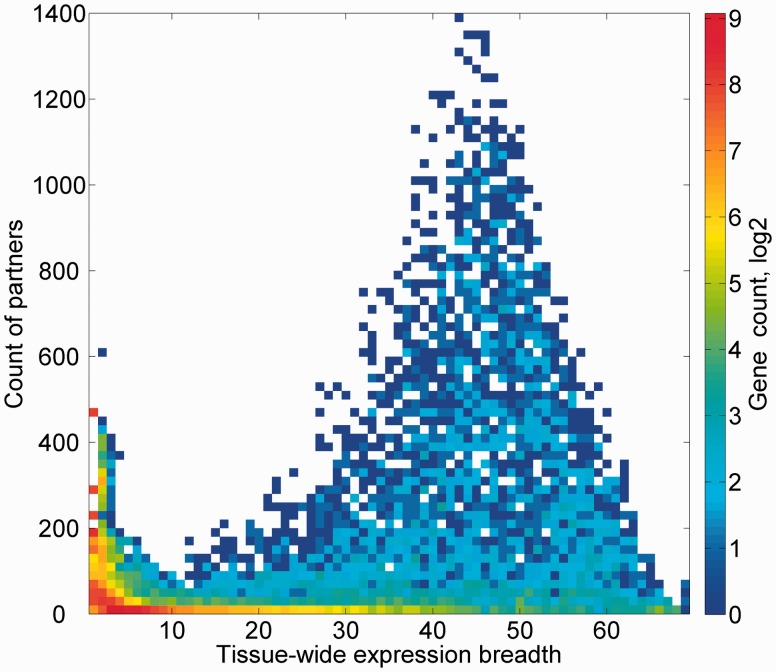
Genes partner count and expression breadth. A heat map showing counts of genes according to bins of partner counts and expression breadth.

We subsequently sought to define a group of expression partners for every relevant gene, based on the pairwise similarity of across-tissue protein abundance patterns. In order to define an optimal expression similarity cutoff, we strived to reconcile between promiscuous partnering and a scarcity of genes with partners. Two different methods for such optimization gave optimal expression similarity rounded cutoff of 0.6 and 0.8, respectively, so a mean of 0.7 was chosen (Supplementary Figures S14A and B). Thus, gene pairs having Pearson’s correlation coefficient of  >0.7 were defined as expression partners. This metric defined 881 932 expression-based gene–gene relationships, each gene having 124 expression partners on average, and the overall gene coverage was 14 264/16 900 genes, with a range of 1–1395 partners (Supplementary Figure S14C). Notably, this expression correlation cutoff is also roughly the line of demarcation of function-sharing enrichment of gene–gene pairs (Figure 9). Another interesting observation is that large expression partner counts (>500) are seen for genes for which expression is seen in 30–60 tissues out of the maximal 69 (Figure 10), i.e. genes that may be crudely defined as housekeeping. The gamut of expression partners for every gene is shown in GeneCards (Figure 7) and will also be available in the GenesLikeMe tool of the GeneCards Suite (33).

We examined a subset of expression partners, focusing on pairs with a well-annotated member and a poorly studied one, as indicated by their GeneCards Inferred Functionality Score (36) (Supplementary Table S11, Figures S10B and S14D). Some of the extreme examples are such that the minimally annotated gene belong to classes of predicted genes, such as those with symbols prefixed with ENSG [from Ensembl (24)], LOC [from Entrez Gene (37)] and containing open reading frames, with practically no functional information; hence, the expression-related pairing could aid in innovative functional insights. An interesting example is the pair *ENSG00000263264*, for which very little annotative information is known, and *RUNX1*, a well-characterized transcription factor that has a role in the development of normal hematopoiesis (25) and is a causative gene for blood diseases (32). Those expression partners are mutually expressed in hematopoietic system cells.

### Proteome-based differential expression

We annotate tissue specificity of genes based on protein abundance. For each gene, the abundance fold change between each tested tissue and the average of all tissues was calculated. We defined genes having fold change of 6 to be differentially expressed in a tissue (Supplementary Figure S15). This created 36 857 gene–tissue annotation pairs, with 26–3142 (average 366) genes being differentially expressed in each tissue, and 0–7 (average 2.2) tissues differentially expressing a given gene (Supplementary Figure S16A and B). The list of tissues in which a gene is differentially expressed is shown in GeneCards, with such information available to its search engine and affiliated tools (Figure 7).

**Figure 11.**
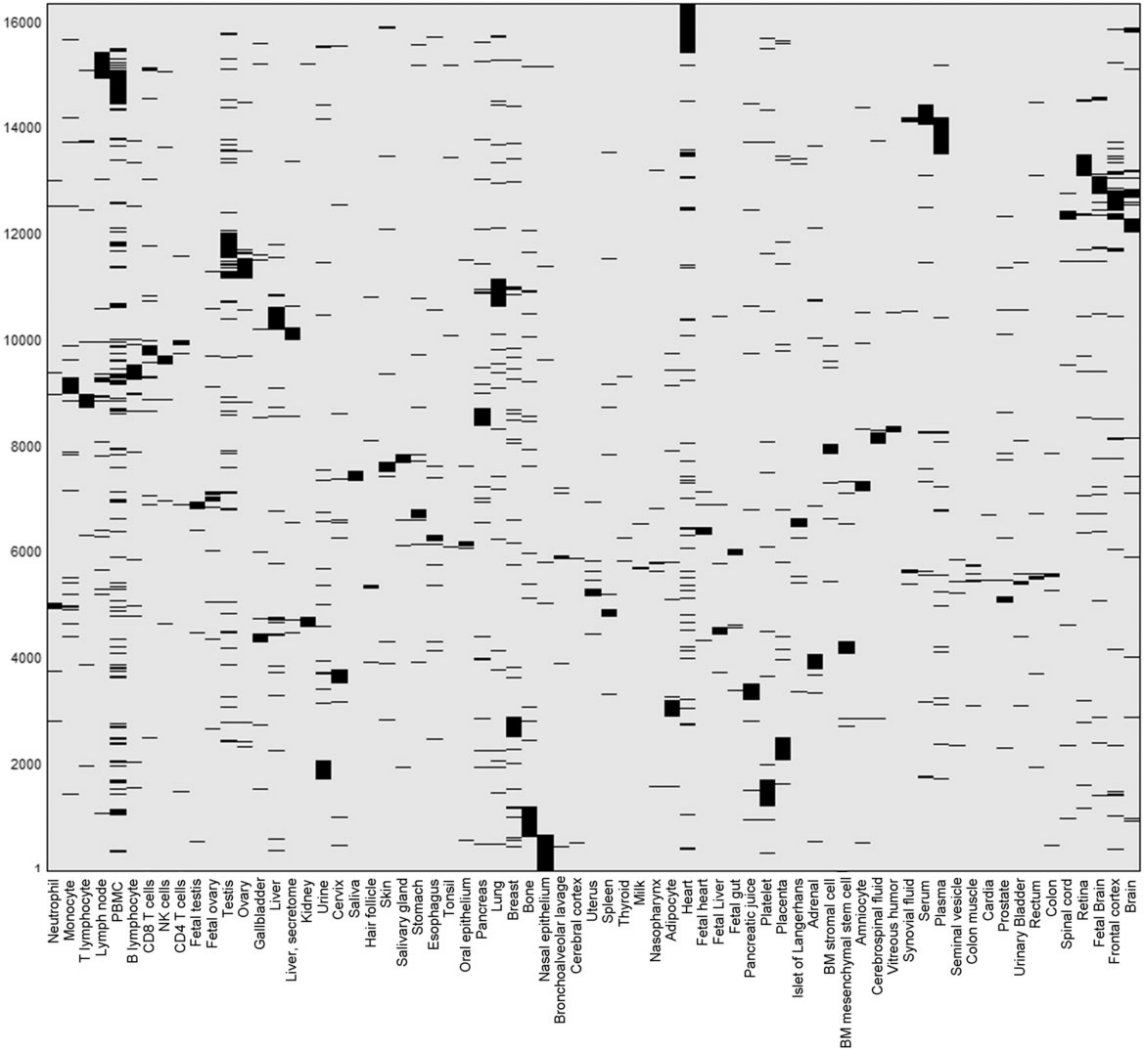
Double hierarchical clustering of the differential expression binary matrix. Analysis included 16 366 genes with differential expression annotation, belonging to the 5839 non-zero patterns. Jaccard coefficient was used as the metric distance.

This differential expression data define a binary matrix with a 16 900-long protein expression column vector for each tissue, and a 69-long tissue expression row vector for each gene (Figure 11). Hierarchical clustering of the gene vectors showed connectivity between anatomically related tissue samples (Supplementary Figure S17). Again, as in Supplementary Figure S4, deviations occur, with a prominent example being the long distance of cerebral cortex from a cluster of other brain tissues (Supplementary Figure S17). This could stem not only from real expression idiosyncrasies but also potentially by experimental error. In total, 5840 distinct across-tissue expression patterns (row vectors) were observed, a very small fraction of the 2^69^ possible ones. This includes all 69 single-tissue patterns (purely tissue-specific genes) and about half of the possible two-tissue patterns (Supplementary Figure S18). Such relatively simple patterns are very frequent among genes, whereby 86% of all genes have patterns with  ≤ 3 tissues. The number of genes showing a given pattern generally declines with the count of tissues in which a gene is differentially expressed (Supplementary Figure S16C). Patterns with higher tissue counts are highly idiosyncratic, with ∼38% of the genes having very rare patterns (populated by ≤ 3 genes), and ∼26% of all genes having a unique pattern not seen in any other gene. We note that the ‘oligo-tissue’ patterns provide a powerful tool for defining gene similarity via expression pattern sharing. In an example, a particular pattern with four tissues (brain, fetal brain, frontal cortex and retina) is seen in 10 genes (*AP3M2*, *CADPS*, *CTNNA2*, *GDAP1*, *GNAO1*, *MAPRE3*, *MYO5A*, *NCAM1*, *PPM1E* and *RAB39B*). Those genes are related to 31 neuronal diseases (Supplementary Table S12).

### Protein to RNA comparisons

Building HIPED provided a large portfolio of human protein abundance profiles. In comparison with RNA expression, we mined GTEx, a comprehensive panel of RNA expression data (26), with RNA-Seq results from 51 types of human samples collected from dozens of individuals per tissue (Supplementary Table S13). We focused on 24 tissue/sample types that are represented in both the protein and RNA data, to facilitate analyses of protein–RNA relationships (Supplementary Table S14). This dataset encompassed 14 155 genes, each with two across-tissue vectors, for protein and for RNA abundances.

**Figure 12.**
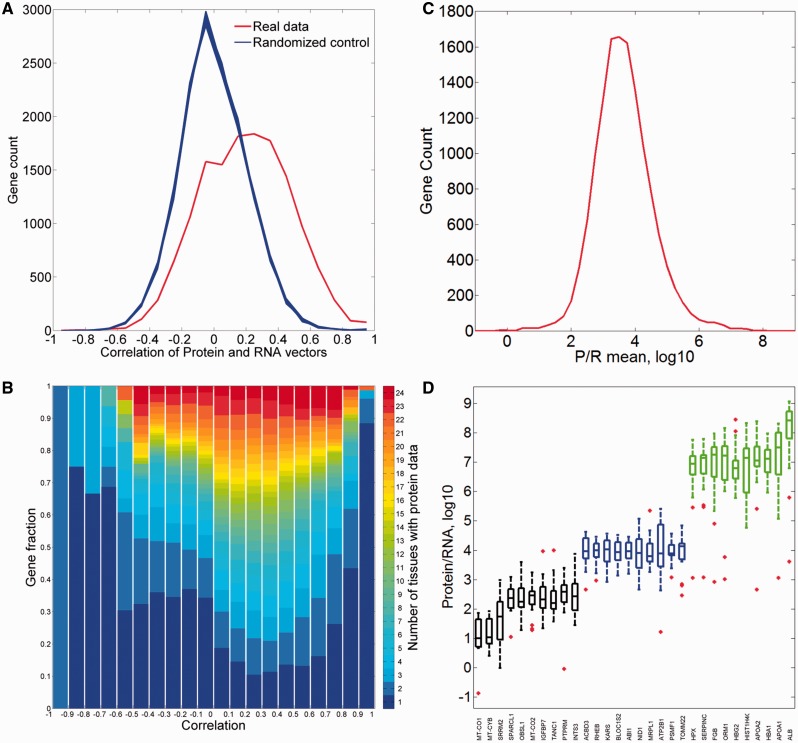
Comparisons of protein and RNA vectors. (**A**) Distribution of Pearson’s correlation coefficient between protein and RNA tissue vectors of every gene in the protein–RNA comparison (red). This distribution is significantly different from the randomized controls (blue, *P*-value of *t*-test <10^−3^). (**B**) Sub-division of each correlation bin using gene fractions according to the number of tissues with protein abundance data. (**C**) Distribution of across-tissue averaged P/R cell copy number ratio of every gene in the protein–RNA comparison. Function enrichment analysis reveals that genes in the upper 10th percentile show a significant enrichment for metabolic and structural functions, while genes in the lower 10th percentile are enriched with signaling and regulation of transcription (Supplementary Tables S15 and S16). (**D**) Box plot of P/R ratios, showing selected 30 genes from distribution peak and both edges.

First, we analyzed the correlation between the protein and RNA vectors (Figure 12A). While the mean Pearson’s correlation coefficient is 0.19, the distribution appears strongly different from that for randomized vectors, with 37% of the genes having a correlation coefficient higher than 0.3. Interestingly, both extremes of the distribution are populated with genes whose expression is strongly tissue specific (Figure 12B).

We subsequently calculated the protein/RNA (P/R) ratios using an absolute scale of cellular copy number for both scales (Figure 12C and D). The ratios have a log-normal distribution, showing a geometric mean of P/R ∼5900, as befits the relatively high molecular count amplification occurring in the translation process. Notably, for genes at the extremes of the distribution, this amplification can be as high as >10^6^ and as low as <10^2^.

**Figure 13.**
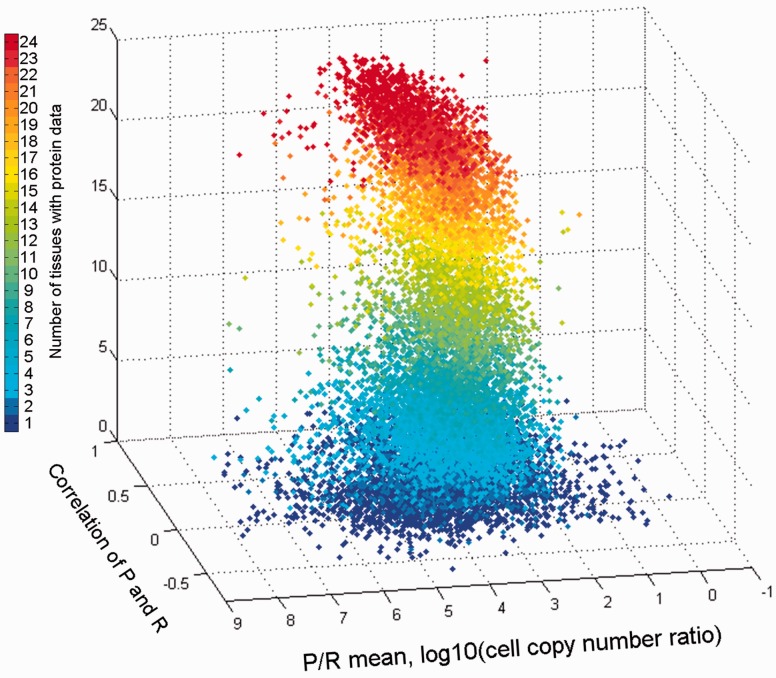
Comparison of gene protein and RNA vectors. A 3D scatter plot of 13 411 genes, using protein–RNA correlation and P/R mean ratio as the *X* and *Y* axes, respectively. The *Z* axis along with the color scale represents the number of tissues with protein data.

The combination of both vectors could help create novel gene-specific signatures. Indeed, different genes reside at different locations of the 3D scatter plot (Figure 13). One geometrical location is that of the default position, namely a strong protein–RNA correlation and average P/R ratio. Other positions represent deviations from this norm, which indicate stronger or weaker translation amplification and/or inter-tissue variations in the P/R ratio. The latter constitutes a deviation from a previous assertion that for many genes the P/R ratio is relatively stable amongst different tissues (5). The 3D position of every gene is being implemented as novel annotation in GeneCards.

## Discussion

### Data integration

The goal of HIPED was to create a unified gene-centric view of protein expression, as obtained from several sources. HIPED integrates only the processed protein abundance values and provides appropriate database links to the original MS-derived data. Our approach strives to provide the user with integrative information on every gene as gleaned from four data sources and hundreds of individual experiments. This is in contrast to an approach whereby data are merely aggregated into one repository, preserving the original individual experiments. Both approaches have been used previously (5, 8–10). Admittedly, our procedure involves certain risks, as the different sources of data use different experimental and analytical methodologies. Examples of inter-experiment differences (38) include (i) different individuals tested; (ii) different part of tissue or cell enrichment protocols employed; (iii) different MS parameters utilized; (iv) different bioinformatic analyses used. An additional challenge to cross-source integration is that the mined sources differ in their gene content (Figure 1D). Thus, a total of ∼12% of the identified genes are reported by only one of the four sources, and only ∼47% are reported by all four sources. This requires judicious procedures for averaging. However, the emerging advantages of integration outweigh the risks, by portraying to the users a clear view of the behavior of each gene product across a variety of anatomical entities.

Of particular relevance is the criticism voiced towards the two genome-wide proteomic studies (4, 5), which we have utilized, questioning their high gene coverage. One critique (39) scrutinized their proteome coverage in the realm of olfactory receptor proteins. Such proteins are nominally not expected to be expressed outside the chemosensory epithelium although evidence to the contrary has been published (40). The conclusions drawn, based on proteomic scrutiny, point to potential shortcomings in spectra interpretation (39, 41, 42). Of note, our Figure 1D does not show genome-wide excess of proteins identified solely in the data mined from one of the questioned sources (5). While the controversy is not fully settled, the data of the appraised sources were subsequently updated (43) and utilized by others (8, 38, 44, 45, 52, 53). We expect that future updates of HIPED will benefit from further progress of proteomic and other OMIC approaches, allowing better annotation of human proteins. HIPED is planned to be updated with up-to-date versions of its sources as a part of GeneCards updates protocols, which take place several times a year.

### Broad tissue set

Analyses that utilize large-scale expression studies for gene annotation, such as determination of gene–gene relationship, comparison of protein and RNA expression, annotation of house-keeping and tissue-specific expression patterns, differ in their tissue content. Some of the tissues/anatomical entities are typically shared by many studies (‘major tissues’), exemplified by liver, heart, brain, testis, ovary, colon, kidney, lung and blood cells (4, 5, 13, 15, 16). Other ‘minor tissues’ include those less often studied, such as fetal samples or tissue sub-compartments. There are even rarer target entities such as bronchoalveolar lavage (a lung biomarker discovery fluid, MOPED) and cardia (a stomach tissue sub-compartment, ProteomicsDB). Our set of 69 normal anatomical entities includes representatives of all the above, and is used as a means of characterizing genes and their mutual relationships. This set of 69 tissues is not inclusive and depends on the particularities of the proteomic sources utilized. Thus, skeletal muscle, agreeably a major tissue, is absent from this set.

Clustering analysis of the protein expression vectors (Supplementary Figure S7) indicated different levels of connectivity, with clusters of close similarity (e.g. nervous, gonads/fetal, gastrointestinal and blood), as well as entities such as certain individual cells and fluids that tend to show very weak inter-tissue similarity. Obviously, obtaining gene–gene relationships benefit from a selection of a subset of maximally orthogonal tissues. At the same time, the inclusion of tissues showing higher similarity would be beneficial by adding fine-tuning. Our comprehensive set of anatomical entities, emerging from the combined effort of the proteome experimentalists, ensures optimal analyses.

HIPED future versions will attempt to further characterize the human proteome. While this study is focused on the normal anatomical entities, some questions regarding the disease and cell line proteomes remain open. One direction to be pursued in subsequent studies is a thorough comparison of the non-normal proteomes with the corresponding normal counterparts. Such efforts might aid the annotation of genes related to the disease phenotypes. Further, they could help identify expression differences between human tissues and their cognate cell lines (Supplementary Figure S8).

### Gene–gene relationships

Many previous analyses report expression profiles for individual genes (4, 5, 8 –10, 15, 26, 46). In this article, we strive to go further by leveraging expression data in order to establish gene expression similarity metrics. Previous attempts to similarly annotate gene–gene expression partners or establish expression-based gene networks, focused on RNA expression datasets and antibody-based proteomics, analyzing across-tissue patterns (13, 14, 18, 47) or time-scale and subcellular localization, as was done in yeast (48). The study described herein is different because it exploits integrated high-throughput MS-based proteomics for this purpose.

We demonstrate that genes tend to be co-expressed much more than randomly expected and form expression groups that populate diverse positions in the expression landscape. A significant aspect of this endeavor rests on the assumption that genes involved in the same function should have correlated expression pattern (49), thus allowing one to ascribe shared functional characteristics to poorly studied genes. In benchmark experiments, we indeed demonstrate links between expression pattern similarities and function. This suggests that the expression similarity scale developed may help define novel gene functional partners.

### Proteomic expression breadth

Expression breadth is used for functional gene annotation, including often-used terms such as housekeeping and tissue-specific genes. The tissue proteomic expression space presented here allows one to define more subtle expression signatures than the two extremes mentioned above, as previously indicated in analyses of mRNA expression (15, 50). Basically, because it is based on a large number of tissues (69), the portrayed expression scale is more graded. What previous reports (16, 17) call housekeeping genes spans a relatively broad range on this scale. We believe that the major reasons for such differences in housekeeping genes annotation are a combination of using RNA expression in previous reports, and the lower number of participating tissues (16 and 43).

Several analyses in this work emphasize tissue breadth- specific signatures. For instance, the expression breadth emerges as the primary PCA axis in the space of genic tissue expression vectors (Figure 6A). Another interesting insight, derived from the expression partner annotation procedure, is that genes with high expression breadth possess many partners.

### Protein–RNA comparisons

Protein and RNA abundances provide two different views of gene expression, and comparing these two scales is not straightforward. Different genes show both widely disparate values of mean protein to RNA ratios (in the range of 10^2^ to 10^6^), as well as a wide range of correlation values for their cross-tissue abundance vectors. In this vein, tissue-centric comparisons previously revealed that although a correlation exists, it is far from being perfect (19, 22, 51). In parallel, it was recently proposed that for most genes, the P/R ratios tend to be similar across different tissues (5, 23). Such behavior is synonymous with there being a high correlation coefficient between protein and RNA levels when analyzed for many tissues. Our data, showing generally modest correlation values, and very poor correlation for quite a few genes, indicate that such a generalization may not always be supported. Further large-scale studies of both RNA and protein abundances performed on identical tissues sources could help better resolve such issues.

### GeneCards

The major findings of the current research were incorporated into the GeneCards database, significantly enhancing its expression section. This makes the recent proteomics progress readily available through a widely used gene-centric database, which hosts HIPED in its relational database tables. The first data layer in the GeneCards web interface is protein abundance histograms across all 69 anatomical entities (Figure 7), joining the existing RNA-based counterpart, recently enhanced with GTEx data (26). This is followed by two textual lists of tissues differentially expressing a given gene, based on protein and RNA data. Finally, the users can view a list of expression partners, i.e. genes with similar expression patterns based on proteomic data. Some of the genes are annotated as ‘elite’ if they are also partnered at the RNA level, shown at the top of the gene list. A third data layer is the provision of HIPED direct and derived data to other GeneCards Suite databases and tools. This includes availability of the textual annotations in the search engine, further allowing users of VarElect, a next-generation phenotyper, affording effective discovery of direct and indirect relationships between variant-harboring genes and disease phenotypes. The protein expression-based similarity metric will soon also be available in the suite’s gene partnering tool GenesLikeMe.

## Supplementary data

Supplementary data are available at *Database* Online.

## References

[1] Mann M. Kulak N.A. Nagaraj N (2013) The coming age of complete, accurate, and ubiquitous proteomes. Mol. Cell, 49, 583–590. 23438854 10.1016/j.molcel.2013.01.029

[2] Legrain P. Aebersold R. Archakov A (2011) The human proteome project: current state and future direction. Mol. Cell Proteomics, 10, M111 009993. 21742803 10.1074/mcp.M111.009993 PMC3134076

[3] Paik Y.K. Jeong S.K. Omenn G.S (2012) The Chromosome-Centric Human Proteome Project for cataloging proteins encoded in the genome. Nat. Biotechnol., 30, 221–223. 22398612 10.1038/nbt.2152

[4] Kim M.S. Pinto S.M. Getnet D (2014) A draft map of the human proteome. Nature, 509, 575–581. 24870542 10.1038/nature13302 PMC4403737

[5] Wilhelm M. Schlegl J. Hahne H (2014) Mass-spectrometry-based draft of the human proteome. Nature, 509, 582–587. 24870543 10.1038/nature13319

[6] Kusebauch U. Deutsch E.W. Campbell D.S (2014*)* Using PeptideAtlas, SRMAtlas, and PASSEL: Comprehensive Resources for Discovery and Targeted Proteomics. Curr. Protoc. Bioinformatics, 46, 13 25 1–13 25 28. 24939129 10.1002/0471250953.bi1325s46 PMC4331073

[7] Vizcaino J.A. Cote R.G. Csordas A (2013) The PRoteomics IDEntifications (PRIDE) database and associated tools: status in 2013. Nucleic Acids Res., 41, D1063–D1069. 23203882 10.1093/nar/gks1262 PMC3531176

[8] Montague E. Janko I. Stanberry L (2015) Beyond protein expression, MOPED goes multi-omics. Nucleic Acids Res., 43, D1145–D1151. 25404128 10.1093/nar/gku1175 PMC4383969

[9] Wang M. Weiss M. Simonovic M (2012) PaxDb, a database of protein abundance averages across all three domains of life. Mol. Cell Proteomics, 11, 492–500. 22535208 10.1074/mcp.O111.014704 PMC3412977

[10] Schaab C. Geiger T. Stoehr G (2012) Analysis of high accuracy, quantitative proteomics data in the MaxQB database. Mol. Cell Proteomics, 11, M111 014068. 10.1074/mcp.M111.014068 PMC3316731 22301388

[11] Safran M. Dalah I. Alexander J (2010) GeneCards Version 3: the human gene integrator. Database (Oxford), 2010, baq020. 20689021 10.1093/database/baq020 PMC2938269

[12] Belinky F. Nativ N. Stelzer G (2015) PathCards: multi-source consolidation of human biological pathways. Database (Oxford), 2015, 10.1093/database/bav006 PMC4343183 25725062

[13] Barshir R. Basha O. Eluk A (2013) The TissueNet database of human tissue protein-protein interactions. Nucleic Acids Res., 41, D841–D844. 23193266 10.1093/nar/gks1198 PMC3531115

[14] Okamura Y. Aoki Y. Obayashi T (2015) COXPRESdb in 2015: coexpression database for animal species by DNA-microarray and RNAseq-based expression data with multiple quality assessment systems. Nucleic Acids Res., 43, D82–D86. 25392420 10.1093/nar/gku1163 PMC4383961

[15] Uhlen M. Fagerberg L. Hallstrom B.M (2015) Proteomics. Tissue-based map of the human proteome. Science, 347, 1260419. 25613900 10.1126/science.1260419

[16] Eisenberg E. Levanon E.Y. (2013) Human housekeeping genes, revisited. Trends Genet., 29, 569–574. 23810203 10.1016/j.tig.2013.05.010

[17] Chang C.W. Cheng W.C. Chen C.R (2011) Identification of human housekeeping genes and tissue-selective genes by microarray meta-analysis. PLoS One, 6, e22859. 21818400 10.1371/journal.pone.0022859 PMC3144958

[18] Szklarczyk D. Franceschini A. Wyder S (2015) STRING v10: protein-protein interaction networks, integrated over the tree of life. Nucleic Acids Res., 43, D447–D452. 25352553 10.1093/nar/gku1003 PMC4383874

[19] de Sousa Abreu R. Penalva L.O. Marcotte E.M (2009) Global signatures of protein and mRNA expression levels. Mol. Biosyst., 5, 1512–1526. 20023718 10.1039/b908315d PMC4089977

[20] Vogel C. Marcotte E.M. (2012) Insights into the regulation of protein abundance from proteomic and transcriptomic analyses. Nat. Rev. Genet., 13, 227–232. 22411467 10.1038/nrg3185 PMC3654667

[21] McManus J. Cheng Z. Vogel C. (2015) Next-generation analysis of gene expression regulation - comparing the roles of synthesis and degradation. Mol. Biosyst., 11, 2680–2689. 26259698 10.1039/c5mb00310e PMC4573910

[22] Schwanhausser B. Busse D. Li N (2011) Global quantification of mammalian gene expression control. Nature, 473, 337–342. 21593866 10.1038/nature10098

[23] Stevens S.G. Brown C.M. (2013) In silico estimation of translation efficiency in human cell lines: potential evidence for widespread translational control. PLoS One, 8, e57625. 23460887 10.1371/journal.pone.0057625 PMC3584024

[24] Cunningham F. Amode M.R. Barrell D (2015) Ensembl 2015. Nucleic Acids Res., 43, D662–D669. 25352552 10.1093/nar/gku1010 PMC4383879

[25] UniProt C. (2014) Activities at the Universal Protein Resource (UniProt). Nucleic Acids Res., 42, D191–D198. 24253303 10.1093/nar/gkt1140 PMC3965022

[26] GTEx Consortium. (2013) The Genotype-Tissue Expression (GTEx) project. Nat. Genet., 45, 580–585. 23715323 10.1038/ng.2653 PMC4010069

[27] Love M.I. Huber W. Anders S. (2014) Moderated estimation of fold change and dispersion for RNA-seq data with DESeq2. Genome Biol., 15, 550. 25516281 10.1186/s13059-014-0550-8 PMC4302049

[28] Mele M. Ferreira P.G. Reverter F (2015) Human genomics. The human transcriptome across tissues and individuals. Science, 348, 660–665. 25954002 10.1126/science.aaa0355 PMC4547472

[29] Milo R. (2013) What is the total number of protein molecules per cell volume? A call to rethink some published values. Bioessays, 35, 1050–1055. 24114984 10.1002/bies.201300066 PMC3910158

[30] Kellis M. Wold B. Snyder M.P (2014) Defining functional DNA elements in the human genome. Proc. Natl. Acad. Sci. U S A., 111, 6131–6138. 24753594 10.1073/pnas.1318948111 PMC4035993

[31] Binder J.X. Pletscher-Frankild S. Tsafou K (2014) COMPARTMENTS: unification and visualization of protein subcellular localization evidence. Database (Oxford), 2014, bau012. 24573882 10.1093/database/bau012 PMC3935310

[32] Rappaport N. Twik M. Nativ N (2014) MalaCards: a comprehensive automatically-mined database of human diseases. Curr. Protoc. Bioinformatics, 47, 1 24 1–1 24 19., 25199789 10.1002/0471250953.bi0124s47

[33] Stelzer G. Inger A. Olender T (2009) GeneDecks: paralog hunting and gene-set distillation with GeneCards annotation. Omics, 13, 477–487. 20001862 10.1089/omi.2009.0069

[34] Ben-Ari Fuchs S Lieder I Stelzer G (2016) GeneAnalytics: An integrative gene set analysis tool, OMICS, in press. 10.1089/omi.2015.0168 PMC4799705 26983021

[35] Safran M. Chalifa-Caspi V. Shmueli O (2003) Human Gene-Centric Databases at the Weizmann Institute of Science: GeneCards, UDB, CroW 21 and HORDE. Nucleic Acids Res., 31, 142–146. 12519968 10.1093/nar/gkg050 PMC165497

[36] Harel A. Inger A. Stelzer G (2009) GIFtS: annotation landscape analysis with GeneCards. BMC Bioinformatics, 10, 348. 19852797 10.1186/1471-2105-10-348 PMC2774327

[37] Maglott D. Ostell J. Pruitt K.D (2011) Entrez Gene: gene-centered information at NCBI. Nucleic Acids Res., 39, D52–D57. 21115458 10.1093/nar/gkq1237 PMC3013746

[38] Higdon R. Kolker E. (2015) Can “normal” protein expression ranges be estimated with high-throughput proteomics? J. Proteome Res., 14, 2398–407. 25877823 10.1021/acs.jproteome.5b00176

[39] Ezkurdia I. Vazquez J. Valencia A (2014) Analyzing the first drafts of the human proteome. J. Proteome Res., 13, 3854–3855. 25014353 10.1021/pr500572z PMC4334283

[40] Feldmesser E. Olender T. Khen M (2006) Widespread ectopic expression of olfactory receptor genes. BMC Genomics, 7, 121. 16716209 10.1186/1471-2164-7-121 PMC1508154

[41] Perez-Riverol Y. Alpi E. Wang R (2015) Making proteomics data accessible and reusable: current state of proteomics databases and repositories. Proteomics, 15, 930–949. 25158685 10.1002/pmic.201400302 PMC4409848

[42] Paik Y.K. Omenn G.S. Overall C.M (2015) Recent advances in the Chromosome-Centric Human Proteome Project: Missing Proteins in the Spot Light. J. Proteome Res., 14, 3409–3414. 26337862 10.1021/acs.jproteome.5b00785

[43] Savitski M.M. Wilhelm M. Hahne H (2015) A scalable approach for protein false discovery rate estimation in large proteomic data sets. Mol. Cell Proteomics, 14, 2394–2404. 25987413 10.1074/mcp.M114.046995 PMC4563723

[44] Wang M. Herrmann C.J. Simonovic M (2015) Version 4.0 of PaxDb: Protein abundance data, integrated across model organisms, tissues, and cell-lines. Proteomics, 15, 3163–3168. 25656970 10.1002/pmic.201400441 PMC6680238

[45] Santos A. Tsafou K. Stolte C (2015) Comprehensive comparison of large-scale tissue expression datasets. PeerJ, 3, e1054. 26157623 10.7717/peerj.1054 PMC4493645

[46] Wu C. Orozco C. Boyer J (2009) BioGPS: an extensible and customizable portal for querying and organizing gene annotation resources. Genome Biol., 10, R130. 19919682 10.1186/gb-2009-10-11-r130 PMC3091323

[47] Pierson E. Consortium G.T. Koller D (2015) Sharing and Specificity of Co-expression Networks across 35 Human Tissues. PLoS Comput. Biol., 11, e1004220. 25970446 10.1371/journal.pcbi.1004220 PMC4430528

[48] Levy E.D. Kowarzyk J. Michnick S.W. (2014) High-resolution mapping of protein concentration reveals principles of proteome architecture and adaptation. Cell Rep., 7, 1333–1340. 24813894 10.1016/j.celrep.2014.04.009

[49] Lopez-Kleine L. Leal L. Lopez C. (2013) Biostatistical approaches for the reconstruction of gene co-expression networks based on transcriptomic data. Brief Funct. Genomics, 12, 457–467. 23407269 10.1093/bfgp/elt003

[50] Yanai I. Benjamin H. Shmoish M (2005) Genome-wide midrange transcription profiles reveal expression level relationships in human tissue specification. Bioinformatics, 21, 650–659. 15388519 10.1093/bioinformatics/bti042

[51] Nagaraj N. Wisniewski J.R. Geiger T (2011) Deep proteome and transcriptome mapping of a human cancer cell line. Mol. Syst. Biol., 7, 548. 22068331 10.1038/msb.2011.81 PMC3261714

[52] Higdon R. Kolker N. Picone A. (2004) LIP index for peptide classification using MS/MS and SEQUEST search via logistic regression. OMICS: JIB, 8, 357–369. 10.1089/omi.2004.8.357 15703482

[53] Montague E. Stenberry L. Higdon R (2014) MOPED 2.5—An integrated multi-omics resource: Multi-omics profiling expression database now includes transcriptomics data. OMICS: JIB., 18, 335–343. 10.1089/omi.2014.0061 PMC4048574 24910945

